# Borderline Tuberculoid Hansen Disease Presenting as Chronic Macrocheilia

**DOI:** 10.4269/ajtmh.21-0632

**Published:** 2021-10-11

**Authors:** Anusuya Sadhasivamohan, Palaniappan Vijayasankar, Kaliaperumal Karthikeyan

**Affiliations:** Department of Dermatology, Venereology and Leprosy, Sri Manakula Vinayagar Medical College and Hospital, Pondicherry, India

A 40-year-old woman presented with an ill-defined, edematous, soft, nontender plaque of size 3.75 cm × 1.5 cm over the right side of upper lip (Figure 1). The patient was asymptomatic, except for mild paresthesia. The lesion had an insidious onset, with gradual progression over 3 years. There was no history of any injections or trauma to the lips. Her past medical and family history was unremarkable. There were no other significant findings in skin and oral mucosa. Sensation over the lesion was preserved. Neurological examination did not reveal any nerve thickening. Systemic examination was found to be normal.

**Figure 1.**
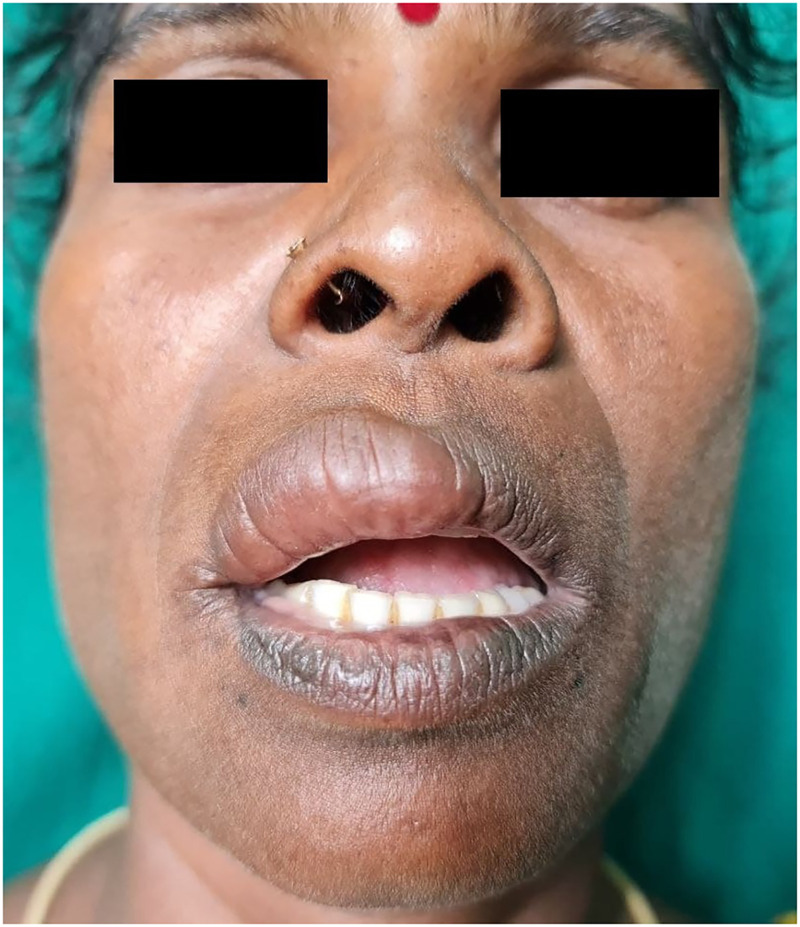
Ill-defined, edematous, soft, nontender plaque of size 3.75 cm × 1.5 cm over the right side of the upper lip. This figure appears in color at www.ajtmh.org.

A differential diagnosis of leprosy and cutaneous tuberculosis was considered because of their endemicity in our region. Mantoux test was negative. A punch biopsy was taken from the lesion. In the dermis, multiple noncaseating epithelioid granulomas admixed with lymphohistiocytic infiltrates and Langhans giant cells were seen around the adnexa and neural bundles (Figure 2). The Fite-Faraco stain (special stain for *Mycobacterium leprae*) was positive, with a bacillary index of 1+ (Figure 3). Based on the clinico-histopathological correlation, a diagnosis of Borderline Tuberculoid Hansen was made. The patient was started on WHO multibacillary-multidrug therapy (MB-MDT). The patient showed partial resolution of the swelling after 6 months of treatment and is under follow-up.

**Figure 2.**
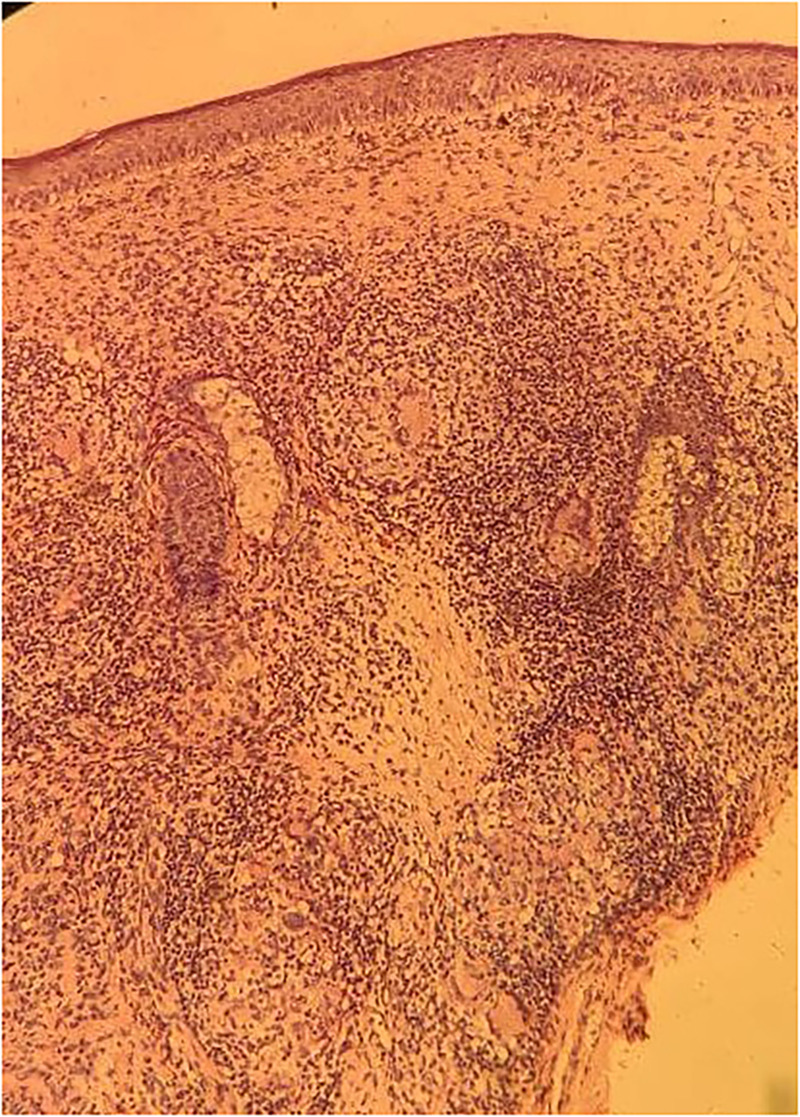
Multiple noncaseating epithelioid granulomas with lymphohistiocytic infiltrates and Langhans giant cells, around the adnexa and neural bundles of dermis (H&E, ×10 magnification). This figure appears in color at www.ajtmh.org.

**Figure 3.**
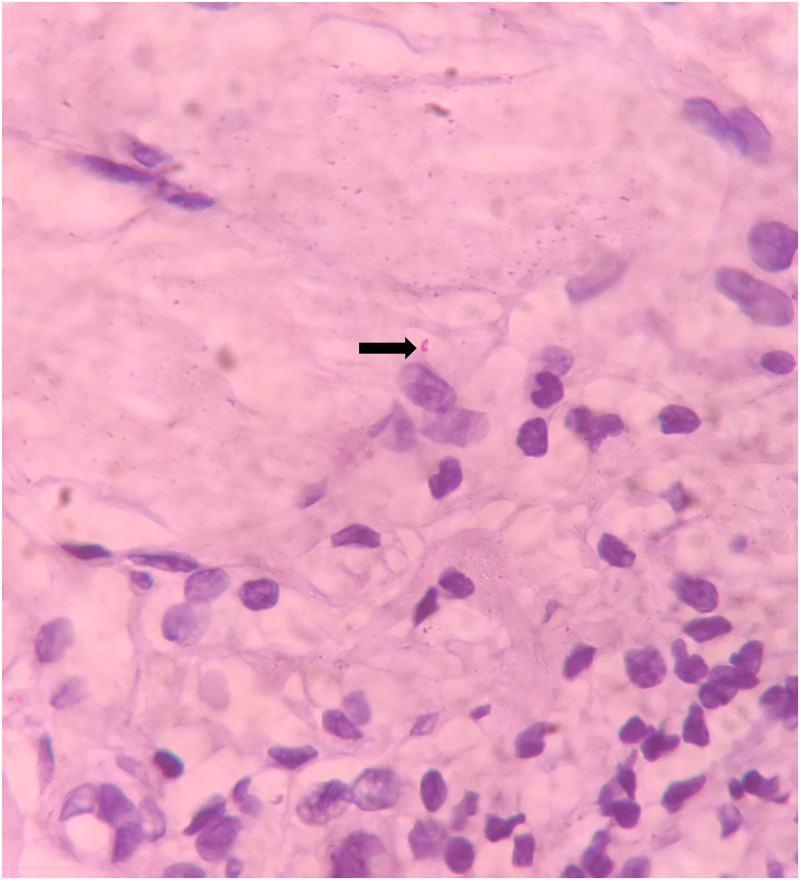
Fite-Faraco stain showing occasional bacilli (arrow), with a bacillary index of 1+ (×100 magnification). This figure appears in color at www.ajtmh.org.

The oral cavity involvement is about 20–60% in lepromatous leprosy whereas it is rare in tuberculoid and borderline tuberculoid spectrum of the disease.^1^ It is usually asymptomatic in nature with slow progression.^2^ The epithelioid granulomas in leprosy block the lymphatic channels, thereby producing swelling of the lips.^1^ It can present as microstomia, macrocheilia, or flat-topped nodules thereby producing severe cosmetic impairment.^2^

The differential diagnosis of chronic macrocheilia includes infections like cutaneous tuberculosis, Hansen disease, syphilis, leishmaniasis, rhinoscleroma, histoplasmosis, and post-odontogenic infections. The noninfectious differential diagnosis such as cheilitis granulomatosa, Melkersson– Rosenthal syndrome, sarcoidosis, Crohn’s disease, amyloidosis, and foreign body reaction should also be considered.^3^^,^^4^

Hansen disease solely presenting as chronic macrocheilia is extremely unusual.^5^ A high index of suspicion for Hansen disease is needed for any chronic macrocheilia in high-endemic areas.

## References

[1] Rao R Kaur GJ Rao AC Chandrashekar B Rao L Handattu S, 2013. Borderline leprosy masquerading as cheilitis granulomatosa: a case report. Lepr Rev 84: 95–99. 23741887

[2] Pallagatti S Sheikh S Kaur A Aggarwal A Singh R, 2012. Oral cavity and leprosy. Indian Dermatol Online J 3: 101–104. 23130281 10.4103/2229-5178.96700 PMC3481884

[3] Van der Waal R Shulten E Van de Scheur MR Wauters I Starink TM Van der Waal I, 2001. Cheilitis granulomatosa. J Eur Acad Dermatol Venereol 15: 519–523. 11843210 10.1046/j.1468-3083.2001.00353.x

[4] Błochowiak KJ 2018. Selected presentations of lip enlargement: clinical manifestation and differentiation. Adv Dermatol Allergol 35: 18–25. 10.5114/ada.2018.73160 PMC5872243 29599668

[5] Handa S Saraswat A Radotra BD Kumar B, 2003. Chronic macrocheilia: a clinico-pathological study of 28 patients. Clin Exp Dermatol 28: 245–250. 12780703 10.1046/j.1365-2230.2003.01284.x

